# Depression and Suicidal Ideation in a Sample of Malaysian Healthcare Workers: A Preliminary Study During the COVID-19 Pandemic

**DOI:** 10.3389/fpsyt.2021.658174

**Published:** 2021-04-30

**Authors:** Hajar Mohd Salleh Sahimi, Tuti Iryani Mohd Daud, Lai Fong Chan, Shamsul Azhar Shah, Farynna Hana Ab Rahman, Nik Ruzyanei Nik Jaafar

**Affiliations:** ^1^Department of Psychiatry, Hospital Canselor Tuanku Muhriz, National University of Malaysia, UKM, Kuala Lumpur, Malaysia; ^2^Department of Psychiatry, Faculty of Medicine, National University of Malaysia, UKM, Kuala Lumpur, Malaysia; ^3^Department of Community Health, Faculty of Medicine, National University of Malaysia, UKM, Kuala Lumpur, Malaysia

**Keywords:** suicidal ideation, health-care workers, early phase, COVID-19 pandemic, Malaysia

## Abstract

**Objective:** The burden of suicidal behavior is anticipated to increase as a sequela of the COVID-19 pandemic. However, there is limited evidence on suicidal behavior among healthcare workers, an at-risk population. Our study aimed to investigate suicidal ideation in terms of the rate and associated factors in a sample of Malaysian healthcare workers during the early-phase of the COVID-19 pandemic.

**Methods:** A subpopulation analysis (*N* = 171) was conducted within a larger, nation-wide cross-sectional study of Malaysian healthcare worker psychological distress from March 18–21, 2020. Current suicidal ideation was measured with item 9 of the Patient Health Questionnaire-9 (PHQ-9). The following independent variables were assessed: socio-demographic profile, occupation and service-related factors, health-anxiety (Health Anxiety Inventory, HAI), lifetime anxiety disorder and severity of depression (PHQ-9).

**Results:** The proportion of healthcare workers with current suicidal ideation (19/171) and clinical depression (17/171) were 11.1 and 9.9%, respectively. Multivariable analysis showed that clinical depression was the most significant factor associated with current suicidal ideation (*p* < 0.001, OR = 55.983, 95% CI = 9.015–347.671) followed by mild (subthreshold) depression (*p* = 0.001, OR = 115.984, 95% CI = 2.977–85.804). Service duration of more than 10 years was associated with significantly less suicidal ideation (*p* = 0.049, OR = 0.072, 95% CI = 0.005–0.993).

**Conclusions:** Depression (subthreshold and especially within the clinical range) and early-career status (<10 years in service) may be target areas of early intervention for reduction of suicidal ideation amongst healthcare workers who have served during the COVID-19 pandemic. Further research is warranted to elucidate specific occupational stressors related to COVID-19 work conditions to tailor appropriate suicide preventive strategies in this population.

## Introduction

Suicidal behavior has been projected to increase globally (1) as a sequela of the anticipated mental health crisis stemming for the COVID-19 pandemic. The mental health needs of frontline healthcare workers (HCWs) have been highlighted as a global priority (2). Medical-related occupational groups have been established as an at-risk population for suicide. A pre-pandemic meta-analysis showed that physicians who are female, United States-based, and in disciplines such as anesthesia, psychiatry, general practice, and general surgery may have higher suicide risk compared to the general population (3).

The myriad stressors faced by HCWs worldwide in their personal and professional spheres are currently compounded by unprecedented challenges due to the COVID-19 pandemic. A meta-analysis showed that the mental health impact of the COVID-19 pandemic on healthcare workers was more severe compared to the general population (4). Significant levels of stress, depression, anxiety, psychological distress, post-traumatic stress symptoms, poor sleep quality, and insomnia have been demonstrated in this frontline population. Majority (92%) of the meta-analyzed studies were conducted in China, with the rest from Iran, Italy, Singapore, and Vietnam (4). Spanish and American healthcare workers' high levels of psychological distress were significantly correlated with fears of being infected or infecting others, especially family and friends, and clinical challenges i.e., perceived uncertainty/lack of control and inadequacy of PPE (5, 6).

Nepalese healthcare workers who felt stigmatized, had a history of receiving psychiatric medication, and reported insufficient preventive measures at work seemed to be at higher risk of experiencing mental health problems (7). Among a sample of Malaysian HCWs, depression, stress, and anxiety were shown to be significantly associated with being unmarried, experiencing fear and uncertainties surrounding COVID-19 infection risk via work-exposure to COVID-19 patients, and being in communities with high transmission rates (8). Potential psychological buffers for HCWs include perceived social recognition (5), perceived peer support and having more than three children (8).

Published systematic research in terms of the pandemic's effect specific to suicidal behavior (ideation, non-fatal and fatal attempt) in this high-risk population (HCWs) is limited. Anecdotal reports have linked the recent suicide deaths amongst frontline HCWs with the collective experience of fear, hopelessness, helplessness, and moral injury in the line of duty during the COVID-19 pandemic (3, 4). These authors speculated that multiple factors contributed to HCW suicide, such as social isolation from support networks and the experience of vicarious traumatization when caring for dying patients. In addition, the perceived risk of infection and transmission of the SARS-COV, compounded by uncertainties surrounding equitable allocation of personal protective equipment (PPE), were postulated to have increased HCW suicide risk. On the contrary, an online survey of 834 HCWs and 2,554 general population participants in Bangladesh did not show any significant association between patient care or PPE-related factors with suicidal behavior (ideation, intent, plan, and attempt) (9). Anxiety is a known risk factor for suicidal behavior (10). The potential impact of health anxiety on the behavioral response to COVID-19 has been highlighted by researchers (11). Therefore, the relationship between suicidal behavior and health anxiety pertaining to HCWs' fear of contracting SARS-CoV-2 is an important area for further research. In terms of gender, female HCWs seemed to be at higher risk of suicidal behavior compared to their male counterparts during this current pandemic (9).

With regards to the spectrum of suicidal behavior, it is important to identify risk factors of suicidal ideation which can be distinct from predictors of more severe spectrum of suicidal behaviors (planned, non-fatal, and fatal attempts) (12–15). Though suicide is a devastating fatal outcome, it is a rare occurrence relative to suicidal ideation or suicide attempts (3, 16). A meta-analysis showed that 17% of physicians experienced suicidal ideation while one percent had previously attempted suicide. Identification of HCWs with suicidal ideation is clinically relevant for implementation of early intervention and prevention of suicide (15). The evidence-base is currently mixed on whether there is a definite increase in suicidal behavior around the world as a sequela of the current pandemic (17). A significant knowledge gap exists in terms of the burden of suicidal ideation and its related factors amongst HCWs within the context of the COVID-19 pandemic, especially in low and middle income (LMIC) countries.

Therefore, the objectives of this study are to investigate the rate of suicidal ideation and the association between suicidal ideation with the following factors: socio-demographic profile, depression, health anxiety and lifetime anxiety disorder, and occupation and service-related factors, amongst a heterogenous and culturally diverse sample of Malaysian HCWs during the early phase of the COVID-19 pandemic.

## Method

### Study Design, Setting and Population

This paper is based on a subpopulation analysis of 171 consecutive participants whereby data on depression and suicidal ideation were available from a larger (*N* = 763) nationwide, cross-sectional study on psychological distress among Malaysian HCWs; during the early phase of the COVID-19 pandemic. Ethics approval was obtained from the Medical Research and Ethics Committee, MREC: NMRR-20-1036-53864 and The National University of Malaysia Ethics Committee, UKM: FF 2020-130). According to the Malaysian Ministry of Health (MOH), frontliners are defined in the context of the COVID-19 pandemic as “individuals with high risks of contracting and transmitting Covid-19 infection i.e., those who are directly exposed to an infected individual, an individual at high risk of being infected, a patient sample or an environment that has the potential to be the source of the infection.” Healthcare workers (HCWs) are categorized by the Malaysian health ministry as frontline essential workers during the pandemic (18). This population comprises of clinicians who are involved directly in providing medical treatment to patients as well as HCWs who are involved in patient care in healthcare facilities i.e., handling of patients' specimens, public health control on the field etc. Thus, our study's operational definition of a health care worker in the frontline of the COVID-19 pandemic includes physicians and clinical medical trainees/students, dental practitioners, pharmaceutical officers, science officers, nurses, occupational/physiotherapists, paramedics, X-ray technicians, medical laboratory technicians, healthcare treatment assistants, public health assistants in government and private healthcare settings (18, 19).

Malaysia is a developing, upper-middle-income country in Southeast Asia with a population of 32 million (20). The health care system consists of 154 government hospitals, 210 private hospitals, 1,090 government health clinics, and 7,718 private clinics. Twenty-seven COVID hospitals were designated for admissions of patients under investigations (PUI) or confirmed case of COVID-19 in Malaysia during the pandemic (21). This study was conducted from 18 March 2020 until 21 March 2020. The study start date coincided with the commencement of the Malaysian Movement Control Order (MCO), which is synonymous with a national lockdown in other countries. Malaysia reported the first case of COVID-19 on 25 January 2020. Our study period captured the exponential rise of COVID-19 in Malaysia with cases increasing from 238 (zero mortality) to 1,183 cases (4 deaths) on 14 and 21 March 2020, respectively (22–24). At that time, Malaysia ranked the highest in South-East-Asia (SEA) and 18th globally with regards to the total number of COVID-19 cases (25). The spike in COVID-19 cases in Malaysia and significant spread to neighboring SEA countries was attributed to a cluster originating from a 4-day religious mass gathering with 16,000 people (including 1,500 foreign participants) held from 27 February to 1 March 2020 in Malaysia (26).

All HCWs working in Malaysia were invited to participate in the study. The inclusion criteria for this study include (i) aged between 18 and 60 years-old and (ii) able to read and write in the English language. Data collection was done through an online survey (Google Form). Advertisements for recruitment were sent or posted via email, social media platforms (e.g., WhatsApp, Twitter, etc.) among the networks of the research team, and via organizational/institutional email listserv/mailing lists/websites. The online survey Google Form included a study information sheet, informed consent form, declaration of anonymity and confidentiality as well as information on how to access crisis helplines. Participants were also given the option of including their contact information if they agreed to be contacted and offered online psychological support. Eligible Malaysian health care workers who gave informed consent were subsequently directed to the self-report questionnaire section in the online survey.

### Measures

The self-report questionnaire section consists of four components: socio-demographic and occupational/service-related data, Health Anxiety Inventory (HAI), presence or absence of lifetime anxiety disorder, and severity of depression (PHQ-9).

#### Socio-Demographic and Occupational/Service-Related Data

The following data were collected from respondents: age, gender, ethnicity, educational level, marital status, type of HCW occupation (physician/non-physician), working hours, and duration of service. Self-reported data on the absence or presence of lifetime anxiety disorder was also collected.

#### Health Anxiety Inventory (HAI)

For this study, the very-short version of HAI was used. This screening instrument assessed health anxiety independently of physical health status with 14 items. Items assess worries about health, awareness of bodily sensations or changes, and feared consequences of having an illness. The very-short version HAI has demonstrated good reliability, criterion validity, and sensitivity to treatment (27). A cut-off point of ≥18 signifies hypochondriasis, and a cut-off point of ≥15 represents high health anxiety (28).

#### Patient Health Questionnaire (PHQ-9)

The PHQ-9 is a self-administered questionnaire which measures the frequency of symptoms of depression using nine items on a 4-point Likert-scale ranging from 0 (not at all) to 3 (nearly every day). It has demonstrated good construct validity (29). A total score ranging from 0 to 27 is obtained by the summing of all item scores. A Malaysian version of the PHQ-9 score ≥10 had a sensitivity of 87% specificity of 82% for detecting clinical depression (major depression according to the DSM-IV: Diagnostic and Statistical Manual of Mental Disorders) in a primary care setting (30). For item 9 of the PHQ-9 (suicidal ideation), respondents reporting “not at all” will be scored 0 (“no” for current suicidal ideation in the past 2 weeks), while respondents reporting “several days,” “more than half of the days,” and “nearly every day” will be scored 1 (“yes” for current suicidal ideation in the past 2 weeks). Previous research showed that respondents who reported suicidal ideation in PHQ-9 had a significantly increased risk of non-fatal or fatal suicide attempt than those who did not have suicidal ideation (31).

### Statistical Analysis

Data was analyzed using STATA software, version 12.0. Descriptive data were generated for all variables. Bivariate analysis was performed using chi-squared and *t*-tests. The dependent variable of suicidal ideation (PHQ item 9) was analyzed as a dichotomous (yes/no) variable. The following variables were analyzed as categorical independent variables: gender, relationship status (single vs. married), lifetime anxiety disorder (yes/no), ethnicity (Malay/non-Malay), education level (low: diploma level and below/high: degree level and above), occupation (physician/non-physician), severity of depression (PHQ-score range: 0-4 = minimal, 5–9 = mild, ≥10 = moderate to severe (clinical depression), working hours per week (<40 h/40 to 50 h/> 50 h) and service duration (years: 1–3/4–6/7–10/>10). Health anxiety (HAI) scores were analyzed as a continuous independent variable (assumption of linearity was met for HAI scores with the outcome dependent variable). Values for 10 cases with missing data on continuous predictors were imputed using median. After deletion of 4 cases with missing values either on gender, relationship status, ethnicity, occupation, and having anxiety disorder, data from 171 respondents were available for analysis. Likelihood ratio test was used to determine which independent variables were included into the multivariable logistic regression model. According to Peduzzi et al. (32), sample size calculation for logistic regression is based on event per variable as *n* = 100+5*i*, where *i* refers to the number of independent variables. As the total sample size in this study was 171, 14 or fewer independent variables were considered adequate to be included in the final regression model.

## Results

Majority of the participants were within the 31–40 years age group, female (70.8%), Malay ethnicity (78.4%), married (62%), and with an educational level of a bachelor's degree or higher (70.2%). Most of the participants were physicians (56.2%), mainly working between 40 and 50 h per week (56.1%), and with a service duration of 7–10 years (36.8%). Eighty-three percent of study participants were healthcare workers in hospitals or healthcare facilities that were designated as COVID-19 treating or screening healthcare facilities. Cronbach alpha for HAI and PHQ were 0.879 and 0.873, respectively. Current clinical depression and lifetime anxiety disorder were present in 9.9% (17/171) and 11.1% (19/171) of participants, respectively. Nineteen out of 171 healthcare workers (11.1%) reported current suicidal ideation in the past 2 weeks. Fifty three percent (10/19) of those with suicidal ideation did not meet criteria for clinical depression (no or subthreshold depressive symptoms). Fifty three percent (9/17) of healthcare workers with clinical depression reported suicidal ideation. There was no multicollinearity between the independent variables. The correlation between the independent variables was 0.45 (<0.8).

From bivariate analysis (Table 1), factors significantly associated with current suicidal ideation were single status (*p* = 0.017), higher levels of health anxiety (*p* = 0.234), and higher severity of depression (*p* < 0.001). Participants with more than 10 years of service duration had a significantly lower rate of current suicidal ideation (*p* = 0.013). Multivariable logistic regression model (Table 2) showed that moderate to severe (clinical) depression was the strongest independent variable significantly associated with current suicidal ideation (*p* < 0.001, OR = 55.983, 95% CI = 9.015–347.671) followed by mild depression (*p* = 0.001, OR = 15.984, 95% CI = 2.977–85.804). Service duration of more than 10 years was associated with significantly less suicidal ideation (*p* = 0.049, OR = 0.072, 95% CI = 0.005–0.993).

**Table 1:** Association between socio-demographic, occupational, and clinical factors with current suicidal ideation among health care workers (*N* = 171).

**Factors**	**Current Suicidal Ideation**				***p*-value**
	**Yes**	**95% CI**	**No**	**95% CI**	
	***n* = 19**		***n* = 152**		
Age, *n* (%)					0.756^*^
18–30	8 (42.11)	(19.13, 65.08)	45 (29.61)	(22.27, 36.94)	
31–40	10 (52.63)	(29.40, 75.86)	93 (61.18)	(53.36, 69.01)	
41–50	1 (5.26)	(−5.13, 15.65)	9 (5.92)	(2.13, 9.71)	
51–60	0 (0.00)	–	5 (3.29)	(0.42, 6.15)	
Gender, *n* (%)					0.812
Male	6 (31.58)	(9.95, 53.21)	44 (28.95)	(21.66, 36.23)	
Female	13 (68.42)	(46.79, 90.05)	108 (71.05)	(63.77, 78.34)	
Ethnicity, *n* (%)					0.768^*^
Malay	16 (84.21)	(67.24, 101.18)	118 (77.63)	(70.94, 84.33)	
Non-Malay	3 (15.79)	(−1.18, 32.76)	34 (22.37)	(15.67, 29.06)	
Relationship status, *n* (%)					0.017
Married	7 (6.60)	(14.40, 52.29)	99 (93.40)	(57.48, 72.79)	
Single	12 (18.46)	(40.71, 85.60)	53 (81.54)	(27.21, 42.52)	
Education level, *n* (%)					0.478
Diploma or lower	7 (36.84)	(14.40, 59.29)	44 (28.95)	(21.66, 36.23)	
Bachelor's degree or higher	12 (63.16)	(40.71, 85.60)	108 (71.05)	(63.77, 78.34)	
Occupation, *n* (%)					0.744
Physician	10(52.63)	(29.40, 75.86)	86 (56.58)	(48.62, 64.54)	
Non-physician	9 (47.37)	(24.14, 70.60)	66 (43.42)	(35.36, 51.38)	
Anxiety, *n* (%)					0.234^*^
Yes	4 (21.05)	(2.08, 40.02)	15 (9.87)	(5.08,14.66)	
No	15 (48.95)	(59.98, 97.91)	137 (90.13)	(85.34, 94.92)	
Working hours per week, *n* (%)					0.584^*^
<40 h	1 (5.26)	(−5.13, 15.65)	22 (14.47)	(8.82, 20.13)	
40–50 h	11 (57.89)	(34.92, 80.87)	85 (55.92)	(47.95, 63.90)	
>50 h	7 (36.84)	(14.40, 59.29)	45 (29.61)	(22.27, 36.94)	
Service duration (years), *n* (%)					0.013^*^
1–3	8 (42.11)	(19.13, 65.08)	21 (13.82)	(8.27, 19.36)	
4–6	3 (15.79)	(−1.18, 32.76)	35 (23.03)	(16.26, 29.79)	
7–10	7 (36.84)	(14.40, 59.29)	56 (36.84)	(29.09, 44.59)	
>10	1 (5.26)	(−5.13, 15.65)	40 (26.32)	(19.24, 33.39)	
Severity of depressive symptoms (PHQ-9), *n* (%)					<0.001
Minimal	2 (10.53)	(−3.75, 24.81)	112(73.68)	(80.76, 66.61)	
Mild	8 (42.11)	(65.08, 19.13)	32 (21.05)	(27.60, 14.50)	
Moderate and severe (clinical)	9 (47.37)	(70.60, 24.14)	8 (5.26)	(1.67, 8.85)	
Health Anxiety (HAI score) (mean ± SD)	20.32 (8.53)		17.16 (6.44)		0.027 ~

Bivariate analysis based on Chi squared test except for * & ~.

* *Fischer exact test*.

*~T-test*.

**Table 2:** Factors associated with current suicidal ideation based on multivariable logistic regression (*N* = 171).

	**S.E**.	***p***	**O.R**.	**95% C.I. for O.R**.	
				**Lower**	**Upper**
Married (vs. single)	0.725	0.948	0.952	0.214	4.233
Service duration (years)					
1–3 (baseline)	–	–	–	–	–
4-6	0.208	0.113	0.211	0.031	1.448
7-10	0.267	0.176	0.297	0.051	1.723
> 10	0.096	0.049	0.072	0.005	0.993
Total HAI score	0.044	0.822	1.010	0.928	1.099
Severity of depression					
Minimal depression (baseline)	–	–	–	–	–
Mild depression	13.705	0.001	15.984	2.977	85.804
Moderate to severe depression	52.163	<0.001	55.983	9.015	347.671

## Discussion

The burden and risk factors of suicidal behavior among health care workers in low- and middle-income countries have not been well-established (33). Our present study showed that 11.1% of healthcare workers in Malaysia reported current suicidal ideation (past 2 weeks) during the early phase of the COVID-19 pandemic and lockdown. Based on Zunin and Meyers' model of phases of disaster, our study period coincides with phase 2, whereby the psychological impact of the initial rapid spread of COVID-19 and environment of unpreparedness can encompass a wide range from shock, overt panic, initial confusion, disbelief, focus on self-preservation and family protection (34). The prevalence rate for current suicidal ideation (past 1 month) was 1.7% in the general population from the 2011 Malaysian National Health Morbidity Survey (35). Malaysian HCWs appear to have a higher rate of suicidal ideation during the early phase of the COVID-19 pandemic compared to available pre-pandemic data among medical students (7%) and the general population in Malaysia (36). Our reported figure of 11.1% is lower than the prevalence of 17% for suicidal ideation among physicians in Dutheil et al. (3) meta-analysis of 61 predominantly Western studies (3). Nevertheless, direct comparison of trends in suicidal behavior (including ideation) among HCWs pre- and post-pandemic is challenging due to limitations of methodological heterogeneity from published data in Malaysia and globally.

Depression is a well-established risk factor for suicidal behavior (ideation, attempt and suicide) among medical physicians and nurses (37, 38). Our study showed that the severity of self-reported depressive symptoms was associated with suicidal ideation, with the clinical range of depression being the most significant factor. Nevertheless, a substantial proportion of HCWs who experienced suicidal ideation (50%) did not meet criteria for clinical depression, i.e., having no or subthreshold depressive symptoms. This finding underscores the importance of understanding other factors that contribute to suicidal ideation beyond depression.

Our study demonstrated that a longer duration of service (more than 10 years) appeared to be protective against suicidal ideation in Malaysian HCWs during the early phase of the COVID-19 pandemic. This finding remained significant after controlling for age, gender, marital status, ethnicity, educational level, type of health care occupation and medical specialty, working hours as well as self-reported clinical depression, health anxiety or other anxiety disorders. Previous research findings have been somewhat mixed in terms of the significance of seniority and suicidal behavior among physicians. The level of seniority was not significantly associated with suicide among physicians in England and Wales (39). Being in practice for more than 30 years seemed to be protective against suicidal ideation in a previous study of 7,905 American surgeons. However, service duration was no longer significant after controlling for burnout and depressive symptoms (40).

Findings from our study echo a signal of increased vulnerability to suicidal ideation in early career compared to senior HCWs. The exact mechanisms underlying the interaction between suicidal behavior and stressors (professional and personal) encountered by HCWs in tandem with their level of work experience; are beyond the scope of this study. Nevertheless, previous research highlighted that physician burn-out can contribute to the complex issue of suicidal behavior in the medical profession (41).

Burn-out has been conceptualized as an occupational phenomenon that is separate from a medical condition (42). This is an important distinction to address in terms of interventional implications and suicide prevention among HCWs. Physicians who were younger in age appear to be more susceptible to burn-out in Malaysian and Chinese populations (43, 44). Being at the frontline of the COVID-19 pandemic increased the risk of severe psychological distress such as insomnia, depressive, and anxiety symptoms among HCWs in China, especially among mid-career compared to senior clinicians (45). In Malaysia, findings from a small descriptive study seemed to suggest that residents experienced more stress, anxiety, and depressive symptoms compared to medical specialists (46). A qualitative study in Egypt identified the following COVID-related stressors faced by psychiatry residents: (i) inadequate precautions to guard against the outbreak, (ii) high exposure risk, (iii) the conflict between family and work, and (iv) the postponement of personal and career plans (47).

Often, clinicians in the early phases of their careers inadvertently end up at the frontline of critical health care provision as part of their training. A study from Singapore reported that COVID-19 frontline HCWs mainly comprised of residents (48). The hierarchical nature of medicine and health care service is also a likely exacerbating factor. Three quarters of the nurses working at the frontline of the COVID-19 pandemic epicenter in Wuhan, China were in the junior category of service (49). The perception of having to practice beyond one's level of competency without adequate supervision may contribute to burn-out in a sample of Australian junior physicians (50).

Data from the general population during the current pandemic have shown that British young adults (18–29 years) have a heightened risk for suicidal ideation compared to middle-aged and older adults (51). In the U.S., young adults (18–29 years) were also a high-risk population for serious psychological distress (52). In that study, stressors included fear of contracting COVID, issues with employment, financial, and educational interruptions. In Spain, financial and occupational stressors such as the perception of lack of supervision, communication, coordination and personnel were associated with suicidal ideation and behavior among health-care workers during the 1st wave of the COVID-19 pandemic. These environmental factors remained significant after accounting for pre-existing mood and anxiety disorders as well as level of exposure to COVID-19 (53). Early- to mid-career healthcare workers in Malaysia may have to contend with a heightened sense of unpreparedness during the initial critical phase of the COVID-19 pandemic and lockdown. This population may be in a more vulnerable position compared to senior, better-resourced colleagues in terms of financial stability, job insecurity and disruptions to career progression and advanced training (e.g., postgraduate, specialty/sub-specialty). Therefore, we postulate that such a scenario may contribute to an increased risk of suicidal ideation in less experienced, junior healthcare workers. Further evidence beyond our study is needed to substantiate these hypotheses.

### Study Implications and Recommendations

In view of depression being a significant factor associated with suicidal ideation, early identification of depression as a target for intervention is a potential suicide preventive strategy (54) among HCWs during this current pandemic. There are unique challenges posed by the current pandemic with regards to the logistics of accessible in-person mental health services. Even before the COVID-19 pandemic, multiple barriers to help-seeking and mental health service pathways existed amongst HCWs. The majority (61%) of a sample of American junior and mid-career physicians acknowledged the benefit of psychiatric services (55). However, these authors showed that only a quarter of that population accessed mental health treatment. Individual and institutional/structural level barriers have been reported, such as lack of time, stigma, fear of loss of confidentiality impacting career advancement and medical licensure, as well as lack of formal HCW-centered wellness programs and counseling/therapy, and treatment cost (55, 56). These issues have been reported to impede the accessibility of much needed mental health care services for HCWs including in Malaysia (57). Solutions to overcome these challenges are strikingly critical in the face of the current pandemic. This is imperative not only to address the obvious mental health care burden and risk of suicide among HCWs, but also to sustain the integrity of health care service systems and optimum patient care as medical errors have been associated with untreated depression in residents (58). Hence, creative implementation of online psychological support and crisis interventions may be a way forward during this pandemic. This would minimize infection risk of in-person mental health service provision, particularly among frontline medical staff as a target population (59). Examples of digital applications (apps) designed specifically to support the mental health of HCWs during this pandemic include TEN – The Essential Network for Health Professionals during COVID-19 (60) and an e-package developed by University of Nottingham (61).

Healthcare workers with less seniority in service appear to be a more at-risk population for suicidal ideation during this pandemic. Current models have recommended pre-frontline deployment training that emphasize resilience building, especially for medical staff that are more junior and less experienced in frontline crisis and disaster health services (59, 62). Previous research during the 2003 Severe Acute Respiratory Syndrome (SARS) epidemic in Taiwan showed that implementation of a SARS prevention program significantly reduced the levels of depression in nursing staff from moderate to minimal or mild (63). The program encompassed infection control and patient care protocols and training, adequate PPE supplies, and access to a mental health clinic specific for HCWs. In order to better understand the risk and protective factors for suicidal behavior amongst HCWs, further quantitative and qualitative research is required to elucidate the trajectory of moral injury (64) and specific COVID-19-related work conditions faced by HCWs, i.e., adequacy of PPE, implementation of training protocols, formal infrastructural and organizational support as well as the impact of social isolation. Future longitudinal studies focusing on the long-term impact and sequelae of the ongoing pandemic on healthcare workers are warranted.

### Limitations

This study is susceptible to type II error due to the relatively small sample size. We acknowledge that our study is limited by cross-sectional design which is unable to determine causality of suicidal ideation found in healthcare workers in the context of the COVID-19 pandemic. The generalizability of findings from this study is limited by the non-random sampling of HCWs in Malaysia. Item 9 of the PHQ was used to assess current suicidal ideation in this study. Previous research has supported the utility of Item 9 of the PHQ-9 as an initial screening measure for risk of suicidal acts at the individual patient-level (31). However, caution has to be exercised with regards to its use as the sole self-reported measure of suicidal behavior without more comprehensive clinical assessment of suicide risk to minimize false-positives or false-negatives (65). Our study was limited to a relatively short duration during the initial critical phase of the COVID-19 pandemic and lockdown in Malaysia. Thus, it was beyond the scope of our current study to detect the mid to long-term effects on HCWs which warrants further research. Previous studies among healthcare workers in heterogenous settings have shown mixed findings in terms of the association between the level of COVID-19 exposure and psychological distress i.e., anxiety (66) suicidal behavior (53). The level of exposure to SARS-CoV-2 was not measured in our study. Nevertheless, the vast majority (83%) of healthcare workers in our study were in designated COVID-19 healthcare facilities. Thus, we have interpreted our study findings in the context of the overall significant risk of exposure to SARS-CoV-2 in our study population, while recognizing that individual level data of exposure to SARS-CoV-2 is a significant confounding variable.

## Conclusions

Our study has highlighted that depression, both at the subthreshold level and particularly in the clinical range, may be associated with suicidal ideation, while a longer service duration is potentially protective against suicidal ideation amongst Malaysian HCWs. Identification and treatment of depression, as well as a focus on early-/mid-career HCWs as a target population for early intervention may be potential areas for suicide prevention amongst HCWs who have been exposed to the COVID-19 pandemic working environment.

## Data Availability Statement

The original contributions presented in the study are included in the article/supplementary material, further inquiries can be directed to the corresponding authors.

## Ethics Statement

The studies involving human participants were reviewed and approved by Medical Research and Ethics Committee. The patients/participants provided their written informed consent to participate in this study.

## Author Contributions

NRNJ (P.I. and grant-holder of this study), HMSS, and LFC conceptualized this study. HMSS and FHAR was involved in the database collection and organization of this study. HMSS, TIMD, LFC, SAS, FHAR, and NRNJ were responsible for data analysis, interpretation of study results, and involved in the writing and review of the final draft of this manuscript. All authors contributed to the article and approved the submitted version.

## Conflict of Interest

The authors declare that the research was conducted in the absence of any commercial or financial relationships that could be construed as a potential conflict of interest.
